# Construction Notes

**Published:** 1895-04-13

**Authors:** 


### CONSTRUCTION NOTES.

### St. Monica's Hospital, Easingwold.

This cottage hospital is planned for six beds, three for either sex, under the supervision of a trained nurse, who acts as matron. The site—on the outskirts of the town—slopes towards the south to one of the principal roads through it. The entrances to the building have, therefore, been arranged from a court enclosed on three sides of the north side of the main block containing the wards and bed-rooms. In addition to servants' and matron's rooms, kitchens, &c., the accommodation comprises a surgery, or operating-room, a private room for matron or superintendent, and a mortuary, ambulance place, laundry, and outbuildings. The brick-work is throughout executed in red brick two inches thick. The gables are in oak, and the roofs are red tiled. The building has been erected and furnished for the benefit of Easingwold and its neighbourhood at the cost of a private benefactor. Messrs. Woodd and Ainstie are the architects.

### New Wing of the West Ham Hospital.

The western extension, the cost of which has been defrayed by Mr. J. Passmore Edwards, consists of three storeys, viz.: Two wards, each accommodating 12 beds. The ground floor is named after Mrs. Eleanor Elizabeth Edwards, wife of Mr. Passmore Edwards. The first floor is named after Mr. J. R. Roberts, a large contributor to the endowment, and contains two private wards. The third floor contains ten cubicles and a common room for the nursing department. In addition to the hospital, and east thereof, extending to the West Ham Lane, are the dispensary buildings for outdoor relief, out of which the building has grown. This is intended only for cases of accidents, there being many factories and large works in West Ham. The original buildings were designed by Messrs. J. L. and A. C. Houston, and erected by Mr. A. Reed at a cost of about £4,500, including the administrative block. The extension is being carried out at a cost of about £3,800 by Mr. Lewis Angell, F.R.I.B.A., the borough engineer, who is acting as honorary architect. Messrs. White and Son are the contractors. The work is now practically completed.

